# Routing Protocol for Heterogeneous Wireless Sensor Networks Based on a Modified Grey Wolf Optimizer

**DOI:** 10.3390/s20030820

**Published:** 2020-02-04

**Authors:** Xiaoqiang Zhao, Shaoya Ren, Heng Quan, Qiang Gao

**Affiliations:** 1 School of Communication and Information Engineering, Xi’an University of Posts and Telecommunications, Xi’an 710121, China; zxq7703@126.com (X.Z.); quan_heng@126.com (H.Q.); 2 Shaanxi Key Laboratory of Information Communication Network and Security, Xi’an University of Posts and Telecommunications, Xi’an 710121, China; 3 College of Mechanical and Electronic Engineering, Northwest Agriculture and Forestry University, Yangling 712100, China; Hillfinder@163.com

**Keywords:** heterogeneous wireless sensor networks, grey wolf optimizer, network lifecycle, energy consumption

## Abstract

Wireless sensor network (WSN) nodes are devices with limited power, and rational utilization of node energy and prolonging the network lifetime are the main objectives of the WSN’s routing protocol. However, irrational considerations of heterogeneity of node energy will lead to an energy imbalance between nodes in heterogeneous WSNs (HWSNs). Therefore, in this paper, a routing protocol for HWSNs based on the modified grey wolf optimizer (HMGWO) is proposed. First, the protocol selects the appropriate initial clusters by defining different fitness functions for heterogeneous energy nodes; the nodes’ fitness values are then calculated and treated as initial weights in the GWO. At the same time, the weights are dynamically updated according to the distance between the wolves and their prey and coefficient vectors to improve the GWO’s optimization ability and ensure the selection of the optimal cluster heads (CHs). The experimental results indicate that the network lifecycle of the HMGWO protocol improves by 55.7%, 31.9%, 46.3%, and 27.0%, respectively, compared with the stable election protocol (SEP), distributed energy-efficient clustering algorithm (DEEC), modified SEP (M-SEP), and fitness-value-based improved GWO (FIGWO) protocols. In terms of the power consumption and network throughput, the HMGWO is also superior to other protocols.

## 1. Introduction

In recent years, with the development of low-power digital circuits and wireless communication technology, wireless sensor networks (WSNs) have been widely used in many fields, such as military reconnaissance, medical aid, urban management, smart home and target tracking [1,2,3,4,5]. WSNs are mainly composed of a base station (BS) and a large number of randomly distributed sensor nodes. The energy of the sensor node is mainly powered by the battery. However, the battery power is very limited, and when the sensor nodes are placed in a complex or harsh environment, it is very difficult to charge or replace the battery [6,7]. Therefore, energy efficiency is the most important and critical task to lengthen the network lifetime in the design of WSN routing protocols [8,9,10,11].

In the routing protocol of WSNs, the most effective way to save the energy of the sensor nodes and lengthen the network life cycle is to design a routing protocol based on clustering [12,13]. The idea of a clustering algorithm is to divide sensor nodes into multiple regions; a region is also called a cluster. A sensor node is selected as a leader in each cluster, which is also known as the cluster head (CH), and the remaining sensor nodes are selected as members of the cluster to send their perceived data information to the nearest CH, which will conduct data information fusion and send data to the BS. The CH will transmit data packets to the BS according to single-hop and multi-hop modes, and the transmission mode is mainly determined by the distance from the CH to the BS.

Many routing protocols based on clustering have been proposed to extend the network life cycle, such as Low-Energy Adaptive Clustering Hierarchy (LEACH) [14], Threshold Sensitive Energy Efficient Sensor Network Protocol (TEEN) [15], Hybrid Energy-Efficient Distributed Clustering (HEED) [16], LEACH-centralized (LEACH-C) [17], a novel Battery-Level Aware Clustering family of schemes (BLAC) [18], etc. However, most clustering routing protocols of WSNs have been mostly based on homogeneous networks [19,20]. Owing to the changes in node resources and network topology, heterogeneous wireless sensor networks (HWSNs) are now more widely used in practice. The HWSNs mainly consider the heterogeneity of energy. The HWSNs routing protocols with heterogeneity of nodes energy such as Stable Election Protocol (SEP) [21], Modified SEP (M-SEP) [22], Prolong-SEP (P-SEP) [23], an Improved version of the Energy Aware Distributed Unequal Clustering Protocol (improved-EADUC) [24], and Distributed Energy-Efficient Clustering Algorithm (DEEC) [25] have been proposed. However, how to more properly utilize the heterogeneity of nodes’ energy to lengthen the network life cycle and increase the network throughput is one of the main problems of the HWSN routing protocol [26,27].

In this paper, we propose a routing protocol for HWSN based on a modified grey wolf optimizer (HMGWO) that further extends the life cycle and balances the energy consumption of the network. Several techniques are used in HMGWO for the selection of CHs as follows. (1) HMGWO defines fitness functions with different weights to select initial clusters according to the heterogeneity of sensor nodes in HWSN. (2) In each initial cluster, HMGWO considers the fitness value of the sensor node as the initial weight in the GWO and dynamically updates the GWO’s weight according to the distance between the wolves and the prey and the coefficient vector so that the selection of CHs is more reasonable. (3) Compared with previously proposed algorithms, the Euclidean distance is used to select the final CHs. HMGWO considers the distance and residual energy in the selection of the final CHs. (4) HMGWO tries to receive the bad cluster set with a certain probability as the initial cluster set in the next iteration; thus, the final selected cluster set is globally optimal in the whole network. As a result, the network lifespan is improved, and the network energy consumption is balanced through the above methods.

The rest of this paper is organized as follows. We describe the related work in Section 2. The GWO is introduced in Section 3. Section 4 gives the details of the proposed algorithm. Section 5 presents the simulation results of the proposed algorithm compared with other algorithms. The results of Section 5 are discussed and analyzed in Section 6. We conclude the paper in Section 7. In addition, the abbreviations used in this paper are listed in Table 1.

## 2. Related Work

With the rapid evolution and development of sensor networks, the routing protocols of WSNs have attracted the attention of many scholars. However, the clustering routing protocol is a more efficient way to reduce the energy consumption of sensor nodes and lengthen the life cycle of networks relative to other typical routing protocols [28,29]. The most famous clustering routing protocol among homogeneous WSNs is the LEACH protocol [14]. In LEACH, the CHs are randomly selected, and the sensor nodes are periodically rotated into CHs. The network energy consumption is evenly allocated to each sensor node to lengthen the network life cycle and enhance the network throughput. However, many improved protocols based on LEACH have been proposed, such as LEACH-B [30], LEACH-C [17], CL-LEACH [31], H-LEACH [32], O-LEACH [33], etc. Vice-CH-enabled centralized cluster-based routing protocol (VCH-ECCR) [19] is a routing algorithm. The VCH-ECCR reduces the clustering frequency by selecting two-stage vice CHs, thereby reducing the overhead caused by clustering. The number of CHs is updated according to the number of surviving nodes in each round in the network. The experimental results illustrate that the protocol is better than that of traditional routing protocols. A fuzzy logic-based energy-efficient clustering for WSNs based on minimum separation distance enforcement between CHs (FL-EEC/D) [20] was proposed. In FL-EEC/D, a fuzzy logic model is proposed for the selection of CHs. When using this model for CH selection, the residual energy, compaction, density, position suitability, and distance from the BS are used as the main parameters to select a better node as the CH. The life cycle of the FL-EEC/D is raised relative to LEACH. The above protocol performs well in homogeneous WSN, but it performs poorly in HWSNs.

On the basis of the above routing protocols of homogeneous WSNs, many routing protocols of HWSNs have been proposed to further lengthen the life cycle of the network [34,35]. A SEP [21] for the HWSNs was proposed by Smaragdakis et al. The different initial energy levels of nodes indicate that this network is a multi-level HWSN. A two-level HWSN is defined by the SEP; namely, advanced nodes and normal nodes. The SEP protocol has different threshold formulas for advanced nodes and normal nodes. Compared with normal nodes, advanced nodes have a higher probability of becoming CHs, which reduces the remaining energy deviation between ordinary nodes and advanced nodes, thus better lengthening the life cycle of the network. An M-SEP [22] for HWSNs was proposed in 2015. The residual energy of the current node and the average energy of the network are considered in the M-SEP protocol, so that nodes with high residual energy have a higher probability of becoming the CH. The transmission rate of packets and the lifetime of the network are improved through this technique. A P-SEP [23] was proposed in 2017. In the selection of CHs of each round, the protocol controls the inherent randomness and deploys the threshold of heterogeneous energy to prevent low-energy nodes from becoming CHs in the next round. This approach results in improved performance in the network’s lifetime. Hosen et al. proposed an energy-centric cluster-based routing protocol (ECCR) [11]. In this protocol, a predefined static cluster is proposed, whose purpose is to reduce the control information overhead in the cluster formation problem. Next, a gatekeeper is introduced, which carries the rank information of nodes with local data. The future cluster director is transferred to the role of the former cluster director of the previous round. This reduces the cost of CH elections over the entire network life cycle, thus extending the network life cycle.

In [25], a DEEC for HWSNs was designed. This protocol is a multi-level heterogeneous network protocol. DEEC chooses the CHs by defining a threshold formula, which is influenced by the remaining energy of the node and the average remaining energy of the network. The period taken for the node to become CH varies according to the remaining energy, so the probability of a high-energy node becoming a CH is higher than that of a low-energy node, which lowers the energy consumption between nodes and lengthens the stable transmission period of the network. An energy efficient clustering protocol to enhance the performance of the HWSN, EECPEP-HWSN [36], was proposed. EECPEP-HWSN was designed as a three-level HWSN, namely including ordinary nodes, advanced nodes, and super nodes. In the process of CH selection, the initial energy of the sensor nodes, hops and residual energy of the sensor nodes during operation are considered. This protocol improves the energy efficiency and stability of HWSN.

In recent years, heuristic and metaheuristic algorithms have been applied to WSNs for theoretical research [37,38,39]. However, many of the above algorithms have also been applied to clustering routing protocols to optimize the selection process of cluster heads, thus improving the network’s life cycle [40,41,42,43,44,45]. Wang et al. proposed a special clustering method called energy centers searching using particle swarm optimization (EC-PSO) [46]. First, the protocol selects CHs by dividing node positions according to geometric methods. Then, the particle swarm optimization (PSO) algorithm is used to search the energy center of the network, and the nearest node to the energy center is selected as the CH. Finally, in order to avoid weak nodes becoming relay nodes, the algorithm proposes a protection mechanism with low energy consumption. By combining the above method, the protocol can effectively lengthen the network lifespan. A Genetic-Algorithm-Based Energy-Efficient Clustering (GAEEC) [47] was proposed. The genetic algorithm (GA) is used twice in the protocol. First, in order to evenly select the number of CHs, the protocol uses GA to divide the network into static clusters by distance. Second, the GA is used in the phase of CH selection. However, the selection of CHs is based on the remaining energy of the current node and the total transmission cost, so that nodes with a larger remaining energy have a higher probability of becoming the CH. Thus, the protocol effectively enhances the performance of the network lifespan. A multilayer hierarchical routing protocol (MLHP) [48] was presented. The algorithm divides the sensor nodes into three levels (normal nodes, advanced nodes, and super nodes). At the level of normal nodes, a centralized selection is proposed in which the BS plays an important role in CH selection. In the area of advanced nodes, a GWO route is presented for data transmission, and a distributed clustering algorithm based on cost function is presented for the level of super nodes. The above algorithm makes the MLHP have a longer network life cycle. In [49], an energy-efficient routing protocol for WSNs based on an improved grey wolf optimizer (FIGWO) was proposed. FIGWO improves the searching ability of the GWO optimal solution by considering the fitness value, and a better distribution and more balanced clustering structure of CHs are guaranteed. The transmission distance of the sensor node is recalculated according to the distance from CH and BS, thus reducing the network energy consumption and prolonging the network lifespan.

## 3. Grey Wolf Optimizer

**Figure 1:**
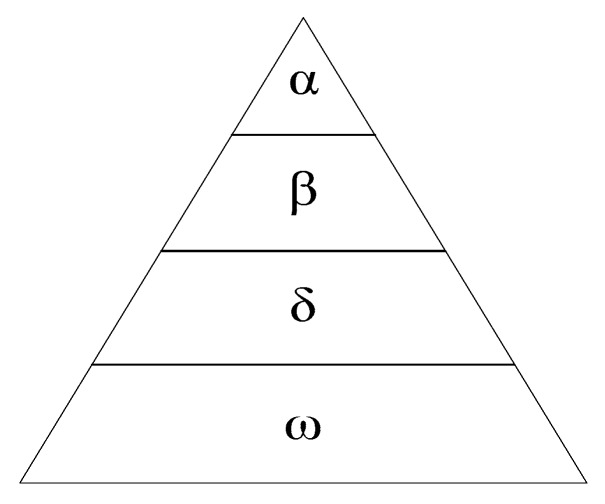
Grey wolf social hierarchy.

The GWO algorithm is designed from the social hierarchy and hunting behavior of grey wolf groups [50]. Grey wolves are gregarious animals with an average population of five to 12. Wolves are classified into four categories: leader wolves (α), the second-best wolves (β), the third-best wolves (δ), and other wolves (ϖ), as shown in Figure 1. Among them, α wolves make decisions about their daily activities, such as how to hunt, when to rest, when to wake up and how to distribute food. The main task of β wolves are not only to assist α wolves in making decisions on the daily behavior of the wolves, but also to report the performance of other wolves to α wolves. δ wolves are the followers of α and β wolves, mainly following the orders of α and β wolves, but can command the lower ϖ wolves. ϖ wolves mainly obey the orders of the higher wolves.

During hunting, the location of the prey is assessed by α, β, and δ wolves, and the remaining wolves calculate the distance between themselves and the prey; then, the wolves encircle the prey. The following is the calculational formula of the wolf’s position

(1) $${\vec{X}}^{t+1}={\vec{X}}_{p}^{t}-\vec{A}\times \vec{D}.$$

(2) $$\vec{A}=2\times \vec{\alpha }\times \vec{r_{1}}-\vec{\alpha }.$$

where ${\vec{X}}^{t+1}$ is the wolf’s position in the ${\left(t+1\right)}^{th}$ iteration, and ${\vec{X}}_{p}^{t}$ is the prey’s position at the $t^{th}$ iteration. $\vec{A}$ is the convergence factor. $\vec{\alpha }$ linearly decreases from 2 to 0 over the course of iterations, and $\vec{r_{1}}$ is a random vector in the range [0,1]. $\vec{D}$ is the distance from the wolves to the prey, and is calculated as follows:

(3) $$\vec{D}=\left|\vec{C}\times {\vec{X}}_{p}^{t}-{\vec{X}}^{t}\right|.$$

(4) $$\vec{C}=2\times \vec{r_{2}},$$

where ${\vec{X}}_{p}^{t}$ and ${\vec{X}}^{t}$ are, respectively, the prey’s position and the wolf’s position in the $t^{th}$ iteration, $\vec{C}$ is the coefficient vectors calculated in (4), and $\vec{r_{2}}$ is a random vector between 0 and 1.

In GWO, the α, β, and δ wolves are thought to be closest to the prey. They also have the most extensive hunting experience, and thus can determine where the prey is located. Therefore, the prey’s location can be calculated as follows

(5) $${\vec{X}}_{p}^{t+1}=\left({\vec{X}}_{\alpha }^{t+1}+{\vec{X}}_{\beta }^{t+1}+{\vec{X}}_{\delta }^{t+1}\right)/3,$$

where ${\vec{X}}_{\alpha }^{t+1}$, ${\vec{X}}_{\beta }^{t+1}$, and ${\vec{X}}_{\delta }^{t+1}$ are the positions of the α wolves, the β wolves and the δ wolves in the ${\left(t+1\right)}^{th}$ iteration, respectively. They are all calculated according to Equation (1). In addition, other wolves will update their positions around the prey, according to Formula (1). At the end of the iteration times, α wolves have the highest fitness value. Therefore, α wolves are considered as the optimal solution of the function. Algorithm 1 describes the GWO pseudo-code.
**Algorithm 1** GWO pseudo-code1: Initialize the grey wolf population $X_{i}\left(i=1,2,\ldots ,n\right)$; 2: Initialize a, A, and C; 3: Calculate the fitness value for each grey wolf; 4: ${\vec{X}}_{\alpha }^{0}=$ the initial position of the leader wolf; 5: ${\vec{X}}_{\beta }^{0}=$ the initial position of the second-best wolf; 6: ${\vec{X}}_{\delta }^{0}=$ the initial position of the third-best wolf; 7: Calculate the initial position of prey (${\vec{X}}_{P}^{0}$) according to Equation (5); 8: **While** (t < Max number of iterations) **9: For** each grey wolf **10:** Update the position of current grey wolf by Equation (1); **11: end For** **12:** Update a, A, and C; 13: Calculate the fitness value for each grey wolf; **14:** Update ${\vec{X}}_{\alpha }^{t}$, ${\vec{X}}_{\beta }^{t}$, and ${\vec{X}}_{\delta }^{t}$; **15:** Calculate the position of prey (${\vec{X}}_{P}^{t}$) according to Equation (5); 16: t=t+1; **17: End while** 18: return ${\vec{X}}_{\alpha }^{t}$.

In GWO, the end of the iteration indicates the best time for the wolves to capture their prey, and that all wolves are closest to their prey. In the routing protocol of the clustering algorithm, the node with the smallest distance in the cluster should be selected as the CH. Therefore, in the proposed algorithm, we assume that the sensor nodes are wolves and the CH is prey.

## 4. Proposed Algorithm

In this section, the routing protocol for HWSN based on the GWO is presented in detail. Lengthening the network life cycle and enhancing the network throughput are the main objectives of the proposed study.

### 4.1. Network Model and Assumptions

In the network model, the following assumptions are made:

1. All nodes are randomly distributed in the two-dimensional geographical area. Once the location is determined, no matter what happens, the location of the nodes will not change;
2. In HWSNs, all sensor nodes are assigned different initial energy levels;
3. The BS is located in the center of the sensing area, and its power is externally supplied;
4. The energy of the sensor node is limited, and the battery cannot be charged;
5. When the sensor node power is exhausted, the node will be considered dead.

### 4.2. Energy Consumption Model

In this paper, the energy consumption model is similar to that used in [51]. In WSNs, nodes are randomly deployed, and the locations of the nodes are not pre-designed. Most of the energy of a node is dissipated due to communication between nodes, depending on the distance between the nodes. Both data transmission and reception consume energy. Therefore, to transmit an (m−bit)-long data packet over the distance d, the required energy is

(6) $$E_{TX}\left(m,d\right)=\{\begin{array}{c} m\times E_{elec}+m\times \varepsilon_{fs}\times d^{2},\;if\;d\leq d_{crossover}\\ m\times E_{elec}+m\times \varepsilon_{mp}\times d^{4},\;if\;d>d_{crossover} \end{array},$$

where $E_{TX}$ is the energy consumed when the node transmits data, $E_{elec}$ is the energy dissipation of the process of transmitting 1 bit of data and the process of receiving 1 bit of data, $\varepsilon_{fs}$ is the coefficient of energy dissipation in the free-space model, $\varepsilon_{mp}$ is the coefficient of energy dissipation in the multi-path attenuation model, and $d_{crossover}$ is the threshold of the transmission distance, which is calculated as

(7) $$d_{crossover}=\sqrt{\varepsilon_{fs}/\varepsilon_{mp}}.$$

However, the energy consumption required by the receiving node to receive an m bit data packet is calculated as follows

(8) $$E_{RX}\left(m\right)=m\times E_{elec}.$$

The energy consumption of CH can be calculated by the above model. The energy consumption of CH mainly includes three aspects: the energy consumption of receiving data packets of member nodes, fusing data and transmitting fusing data to the base station. The calculation formula is as follows:

(9) $$E_{CH}=E_{RX}\left(m\right)\times CM_{num}+m\times E_{DA}\times (CM_{num}+1)+E_{TX}\left(m,d\right)$$

where $CM_{num}$ is the number of member nodes in the cluster and $E_{DA}$ is 1 bit of data aggregation energy cost; m is the packet length.

The energy consumption of the non-CH node is only the energy consumption of sending data to the CH, and its mathematical expression is as follows:

(10) $$E_{non-CH}=E_{TX}\left(m,d\right)$$

The total residual energy in the $r^{th}$ round is calculated as follows:

(11) $$E_{totR}\left(r\right)=E_{totR}\left(r-1\right)-\left(\sum_{i=1}^{CH_{num}\left(r\right)}E_{CH}\left(i\right)+\sum_{j=1}^{N_{alive}\left(r\right)-CH_{num}\left(r\right)}E_{non-CH}\left(j\right)\right)$$

where $E_{totR}\left(r-1\right)$ is the total residual energy in the ${\left(r-1\right)}^{th}$ round, $CH_{num}\left(r\right)$ represents the number of CHs in the $r^{th}$ round, $N_{alive}\left(r\right)$ represents the number of alive nodes in the network in the $r^{th}$ round, $E_{CH}\left(i\right)$ is the energy consumed by the $i^{th}$ CH, and $E_{non-CH}\left(j\right)$ is the energy consumed by the $j^{th}$ non-CH.

### 4.3. Proposed Algorithm

In order to avoid the randomness of the CH selection, a centralized clustering routing protocol is proposed. The stage of CH selection is calculated centrally by BS through the modified grey wolf optimizer (MGWO), and then the result of CH selection is notified to all sensor nodes in the network in the form of broadcast. The phases at which the cluster member joins the cluster and steady-state are the same as in the LEACH [14] protocol. In the first stage of CH establishment, the sensor nodes first send the location and initial energy to the BS, which receives the information and saves it. The BS is given the initial energy of the nodes, and the node’s position is fixed. The energy consumption of the node can be estimated by clustering information of each round, and the energy information of the node in each round can be obtained. Therefore, the nodes do not need to send position and energy information to the BS in each round.

#### 4.3.1. Selection of Initial Clusters

The protocol uses the energy of the node and the distance from the node to the BS as parameters to select the initial clusters of the network, thereby fixing and limiting the number of CHs in each round, so that the number of CHs in each round is even. The use of the distance parameter makes the distribution of CHs in the network more uniform. The following are the rules used for selecting the initial clusters. The set of alive sensor nodes is divided into m (m is the number of desired clusters, which equals N/p, where N is the number of sensor nodes, and p is the portion of CHs) equal subsets according to the ascending result of the fitness value of the sensor nodes. In each subset, the sensor node that is closest to the middle point is selected as the initial CH. Finally, each node is added to the nearest CH according to the Euclidean distance to form the initial cluster. The node’s fitness value is calculated according to the distance from the node to the BS and residual energy:

(12) $$F_{1}=\{\begin{array}{c} \begin{array}{c} a_{1}\times \frac{E_{r}}{E_{i}}+\left(1-a_{1}\right)\times \left(\frac{d_{MAXBS}-d_{toBS}}{d_{MAXBS}-d_{MINBS}}\right),\;E_{r}>0\;\mathrm{and}\;\mathrm{the}\;\mathrm{node}\;\mathrm{is}\;\mathrm{the}\;\mathrm{normal}\;\mathrm{node}\\ \left(1-a_{1}\right)\times \frac{E_{r}}{E_{i}}+a_{1}\times \left(\frac{d_{MAXBS}-d_{toBS}}{d_{MAXBS}-d_{MINBS}}\right),\;E_{r}>0\;\mathrm{and}\;\mathrm{the}\;\mathrm{node}\;\mathrm{is}\;\mathrm{the}\;\mathrm{advanced}\;\mathrm{node} \end{array}\\ 0\;,\;E_{r}\leq 0\; \end{array},$$

where $a_{1}$ is the weight, $E_{i}$ is the initial energy of the node, $E_{r}$ is the residual energy of the node, $d_{toBS}$ is the distance from the node to the BS, and $d_{MAXBS}$ and $d_{MINBS}$ are, respectively, the maximum and minimum distances between a sensor node and the BS.

#### 4.3.2. Modified Grey Wolf Optimizer

The MGWO is used to select the CHs. In the original GWO [50], the prey’s position was calculated based on the average weight of the α, β, and δ wolves. Considering the difference between the residual energy of the node and the distance between the node and the BS, the fitness value of the node is taken as the initial weight in the GWO, which is calculated by Equation (12). Consequently, based on the GWO and formula (12), the initial position of prey is computed as

(13) $${\vec{X}}_{p}^{0}=\omega_{I\alpha }{\vec{X}}_{\alpha }^{0}+\omega_{I\beta }{\vec{X}}_{\beta }^{0}+\omega_{I\delta }{\vec{X}}_{\delta }^{0},$$

(14) $$\begin{array}{c} \omega_{I\alpha }=F_{\alpha }/\left(F_{\alpha }+F_{\beta }+F_{\delta }\right)\\ \omega_{I\beta }=F_{\beta }/\left(F_{\alpha }+F_{\beta }+F_{\delta }\right)\\ \omega_{I\delta }=F_{\delta }/\left(F_{\alpha }+F_{\beta }+F_{\delta }\right) \end{array}\;,$$

where $\omega_{I\alpha }$, $\omega_{I\beta }$, and $\omega_{I\delta }$ represent the initial weights of the α wolf, the β wolf, and the δ wolf, respectively. $F_{\alpha }$, $F_{\beta }$, and $F_{\delta }$ are the first, the second, and the third best fitness values, respectively, which are calculated through Formula (12). The α, β, and δ wolves are the respective nodes corresponding to the three best fitness values.

When the GWO is implemented in the proposed protocol, the fitness value of the node is changed after completing one data transmission, so the weight of the GWO will not change. In order to further improve the global search capability of the GWO, the weights are dynamically updated by vectors D and A. D is the distance between the wolf and its prey, and A is the coefficient vector. D and A are calculated according to Equations (2) and (3). At the ${\left(t+1\right)}^{th}$ iteration, the location of the prey and the weight updating formula are expressed as

(15) $${\vec{X}}_{p}^{t+1}=\omega_{\alpha }^{t+1}{\vec{X}}_{\alpha }^{t+1}+\omega_{\beta }^{t+1}{\vec{X}}_{\beta }^{t+1}+\omega_{\delta }^{t+1}{\vec{X}}_{\delta }^{t+1},$$

(16) $$\omega_{\alpha }^{t+1}=\left({\vec{D}}_{\alpha }^{t+1}\times {\vec{A}}_{\alpha }^{t+1}\right)/\left({\vec{D}}_{\alpha }^{t+1}\times {\vec{A}}_{\alpha }^{t+1}+{\vec{D}}_{\beta }^{t+1}\times {\vec{A}}_{\beta }^{t+1}+{\vec{D}}_{\delta }^{t+1}\times {\vec{A}}_{\delta }^{t+1}\right)\;\omega_{\beta }^{t+1}=\left({\vec{D}}_{\beta }^{t+1}\times {\vec{A}}_{\beta }^{t+1}\right)/({\vec{D}}_{\alpha }^{t+1}\times {\vec{A}}_{\alpha }^{t+1}+{\vec{D}}_{\beta }^{t+1}\times {\vec{A}}_{\beta }^{t+1}+{\vec{D}}_{\delta }^{t+1}\times {\vec{A}}_{\delta }^{t+1}).\;\omega_{\delta }^{t+1}=\left({\vec{D}}_{\delta }^{t+1}\times {\vec{A}}_{\delta }^{t+1}\right)/\left({\vec{D}}_{\alpha }^{t+1}\times {\vec{A}}_{\alpha }^{t+1}+{\vec{D}}_{\beta }^{t+1}\times {\vec{A}}_{\beta }^{t+1}+{\vec{D}}_{\delta }^{t+1}\times {\vec{A}}_{\delta }^{t+1}\right)$$

where ${\vec{X}}_{\alpha }^{t+1}$, ${\vec{X}}_{\beta }^{t+1}$ and ${\vec{X}}_{\delta }^{t+1}$ are the positions of the α wolf, β wolf, and δ wolf in the ${\left(t+1\right)}^{th}$ iteration, respectively. ${\vec{X}}_{\alpha }^{t+1}$, ${\vec{X}}_{\beta }^{t+1}$ and ${\vec{X}}_{\delta }^{t+1}$ can be obtained by Equation (1).

At the end of the iteration, the node closest to the prey is generally selected as the CH. However, the residual energy of the node may not be able to complete the task of the CH, causing the node to die prematurely. Therefore, the nodes that are close to the prey and have more residual energy should be selected as the CH. Here, it is proposed to use the remaining energy of the node and the distance between the node and the prey as parameters of the fitness function to select the CH. The node with the smaller fitness value is more likely to become the CH. The fitness function is as follows:

(17) $$F_{2}=\{\begin{array}{c} \begin{array}{c} a_{2}\times \frac{E_{MAX}-E_{r}}{E_{MAX}-E_{MIN}}+\left(1-a_{2}\right)\times \left(\frac{d_{MAXP}-d_{toP}}{d_{MAXP}-d_{MINP}}\right),\;E_{r}>0\;\mathrm{and}\;\mathrm{the}\;\mathrm{node}\;\mathrm{is}\;\mathrm{the}\;\mathrm{normal}\;\mathrm{node}\\ \left(1-a_{2}\right)\times \frac{E_{MAX}-E_{r}}{E_{MAX}-E_{MIN}}+a_{2}\times \left(\frac{d_{MAXP}-d_{toP}}{d_{MAXP}-d_{MINP}}\right),\;E_{r}>0\;\mathrm{and}\;\mathrm{the}\;\mathrm{node}\;\mathrm{is}\;\mathrm{the}\;\mathrm{advanced}\;\mathrm{node} \end{array}\\ 0\;,\;E_{r}\leq 0\; \end{array},$$

where $a_{2}$ is the weight; $E_{r}$ is the residual energy of the node; $E_{MAX}$ and $E_{MIN}$ are the maximum and the minimum residual energy in the cluster, respectively; $d_{toP}$ is the distance from the node to the prey; and $d_{MAXP}$ and $d_{MINP}$ are the maximum and minimum distance, respectively, between a sensor node and the prey in the cluster.

#### 4.3.3. Selection of the Optimal Cluster Set

According to the clustering algorithm, a network can be divided into multiple clusters, and multiple clusters in the network are called a cluster set. In this study, the selected initial clusters are referred to as the initial cluster set, which is taken as the current optimal cluster set, and the objective function value of the current optimal cluster set is calculated. Each cluster in the current optimal cluster set is randomly changed by the MGWO to generate a new cluster, and a plurality of the new clusters forms a new cluster set; the objective function value of the new cluster set is then calculated. When the value of the objective function of the current optimal cluster set is greater than that of the new one, the new cluster set is accepted as the current optimal cluster set; otherwise, the new cluster set is accepted as the current optimal cluster set in a probabilistic manner. At the end of the iteration, the optimal cluster set is formed. The objective function is defined as

(18) $$F_{3}=a_{3}\times d_{TCH}+\left(1-a_{3}\right)\times d_{TBS}.$$

where $a_{3}$ is the weight, $d_{TCH}$ is the total intra-cluster communication distance, and $d_{TBS}$ is the total communication distance from the CH to the BS. The objective function is designed based on the total inter-cluster communication distance and the total communication distance from the CH to the BS. A smaller objective function value indicates that the CH selection is more reasonable, the CH is optimal in the cluster, and the CH set is also optimal relative to the whole network. The HMGWO pseudo-code is described in Algorithm 2.
**Algorithm 2** HMGWO pseudo-code**Input:** N = number of alive nodes K = number of desired clusters **Output:** A set of K clusters **Steps:** 19: Initialization; 20: Initial CHs selection (described above); 21: Each of the remaining nodes decides to join its nearest CH according to the Euclidean distance; 22: Form the initial clusters; 23: Current optimal cluster set = initial cluster set; 24: Calculate the objective function value of the current optimal cluster set ($F_{opt}$); 25: **While**
$t\leq t_{max}$
**do** 26: **For** all current optimal clusters 27: **If** (the number of cluster members ≥3) 28: Implement the MGWO algorithm (presented above) to select the CHs in each current optimal cluster; 29: **Else** 30: All the nodes are regarded as normal nodes; 31: **End if** **32: End for** 33: Each of the remaining nodes decides to join its nearest CH according to the Euclidean distance; 34: Form the new clusters; 35: Calculate the objective function value of the new cluster set ($F_{new}$); 36: **If** ($F_{new}<F_{opt}$) 37: Current optimal cluster set = new cluster set; 38: $F_{opt}\;=\;F_{new}$; 39: **Else If** (rand()>probability) 40: Current optimal cluster set = new cluster set; 41: $F_{opt}\;=\;F_{new}$; 42: **End if** 43: **End while**

## 5. Simulation Results

To verify its performance, MATLAB software (MathWorks, USA) was used to simulate the HMGWO algorithm. Under the same experimental conditions, the HMGWO algorithm was compared with the SEP, DEEC, M-SEP, and FIGWO algorithms. The main simulation parameters are shown in Table 2.

At the beginning of each round, the BS selects CHs using the algorithm proposed in this article, and each round last for 1 s. In this paper, we used the network lifetime, residual energy and the number of packets received by the BS as the evaluation indexes of the algorithm performance. However, since the HMGWO, SEP, DEEC, M-SEP, and FIGWO protocols are all single-hop routing protocols, the end-to-end delay was not taken into account in the indicator evaluation. The following is an explanation of indicators and nouns.

1. Network lifetime: The period of time between the beginning of the network operation and the death of the first node, which is also known as the network stability period.
2. Network instability period: The time duration between the death of the first node and the death of all nodes.
3. Total residual energy: The sum of the remaining energy of all survival nodes. To facilitate the comparison of the total residual energy, the percentage of the total residual energy is used to represent the total residual energy [10,20], which is calculated as follows:
  (19) $$p_{t}\left(r\right)=E_{totR}\left(r\right)/\sum_{j=1}^{N}E_{i}\left(j\right)$$
  where $p_{t}\left(r\right)$ is the percentage of the total residual energy in the $r^{th}$ round; $E_{totR}\left(r\right)$ is the total residual energy in the $r^{th}$ round; and $\sum_{j=1}^{N}E_{i}\left(j\right)$ is the sum of the initial energies of all the nodes.
4. Residual energy deviation: The deviation between the node with the most residual energy and the node with the least residual energy [46]. The residual energy deviation in the $r^{th}$ round is calculated as follows:
  (20) $$D_{R}\left(r\right)=E_{MaxR}\left(r\right)-E_{MinR}\left(r\right)$$
  where $E_{MaxR}\left(r\right)$ and $E_{MinR}\left(r\right)$ are the maximum and the minimum residual energy of the node in the network the $r^{th}$ round. However, to facilitate the analysis of the simulation results, the residual energy deviation is compared in percentage units.
5. Throughput: The number of data packets received by the BS.

### 5.1. Network Lifetime

**Figure 2:**
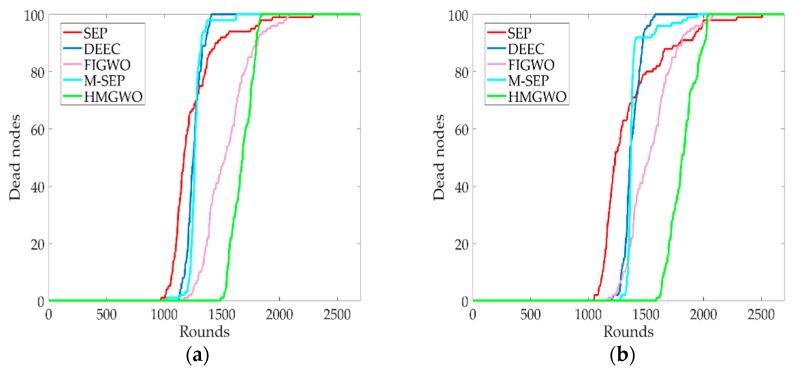
Network stability period with respect to the number of rounds. (**a**) The ratio of the advanced node is 0.1, and the HMGWO network stability period is compared with SEP, DEEC, M-SEP, and FIGWO; (**b**) The ratio of the advanced node is 0.2, and the network stability period of HMGWO relative to SEP, DEEC, M-SEP, and FIGWO.

Figure 2 shows that the HMGWO was superior to other protocols in terms of its network lifespan for different proportions of advanced nodes obtained from the simulation. When the ratio of advanced nodes is α=0.1, it can be seen in Figure 2a that the network lifespan of the HMGWO increased by 55.7%, 31.9%, 46.3%, and 27.0%, respectively, relative to SEP, DEEC, M-SEP, and FIGWO. When the ratio of advanced nodes is α=0.2, it can be seen in Figure 2b that the network lifespan of the proposed algorithm is increased by 51.2%, 32.0%, 24.6%, and 32.8%, respectively, relative to SEP, DEEC, M-SEP, and FIGWO. The main reason for the above results is that the nodes with low residual energies have a low probability of becoming the CH, which avoids the phenomenon of rapid death of the node with low residual energy, thus extending the life cycle of the network.

**Figure 3:**
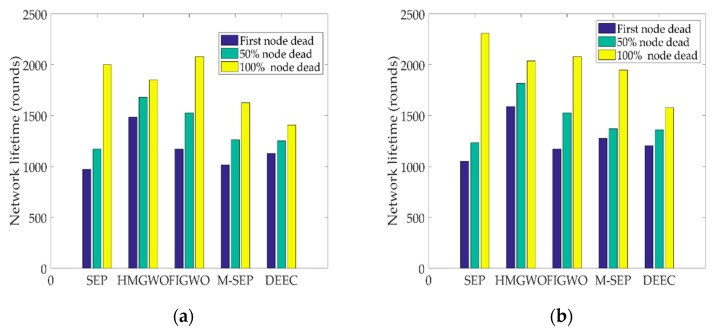
Network lifetime with respect to the number of rounds. (**a**) The network lifetimes of HMGWO, SEP, DEEC, M-SEP, and FIGWO when α is 0.1; (**b**) The network lifetimes of the HMGWO, SEP, DEEC, M-SEP, and FIGWO when α is 0.2.

The simulation results of the network life cycle for when the first node died, 50% of the nodes died and all nodes died are shown in Figure 3 at different proportions of advanced nodes. As can be seen from Figure 3, the death time of the first node was later than that of other protocols. When the number of death nodes reached 50%, the network lifetime of HGWO was significantly extended compared to that of the SEP, FIGWO, DEEC, and M-SEP protocols. However, as can be seen in Figure 3a,b, when all nodes died, the lifetimes of SEP and FIGWO were greater than that of the algorithm proposed in this paper. It can be clearly seen in Figure 3 that the network instability period of the HMGWO was shorter than that of other protocols. The main reason for this is that the HMGWO considers the heterogeneity of nodes and the global optimization ability, such that the energy consumption between nodes is relatively balanced, and the network instability period of the HMGWO is shorter. The specific reasons are discussed in detail in Section 6.

### 5.2. Number of Packets Received by the BS

**Figure 4:**
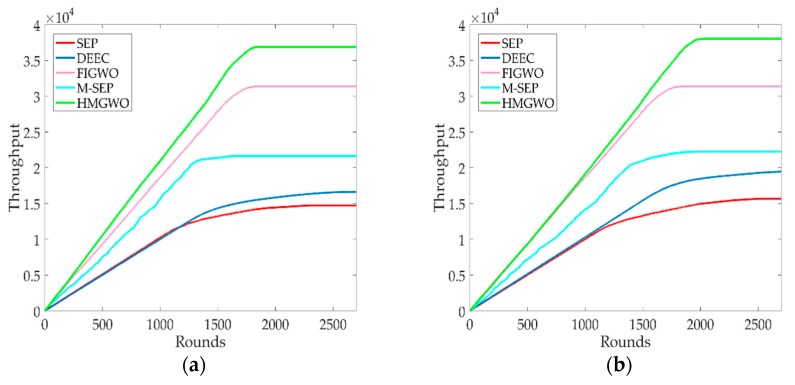
Number of data packets received by the base station. (**a**) The throughput of HMGWO against SEP, DEEC, M-SEP and FIGWO when α is equal to 0.1; (**b**) The throughput of HMGWO is compared to SEP, DEEC, M-SEP and FIGWO when α is equal to 0.2.

Figure 4a,b show the number of data packets received by the BS. When the ratio of advanced nodes is α=0.1, it can be seen from Figure 4a that the network throughput of HMGWO increased by 150.4%, 142.5%, 70.5%, and 17.6%, respectively, relative to SEP, DEEC, M-SEP, and FIGWO. When the ratio of advanced nodes is α=0.2, it can be seen in Figure 4b that the network throughput of the proposed algorithm improved by 142.7%, 124.8%, 70.9%, and 32.8%, respectively, relative to SEP, DEEC, M-SEP and FIGWO. This is because the HMGWO algorithm adopts a more reasonable CH selection mechanism, and the nodes have a longer survival time. When the number of rounds is the same, the number of surviving nodes in the network is higher than that of other algorithms, so more data groups are generated, and the number of data packets received by the BS also increases.

### 5.3. Residual Energy

**Figure 5:**
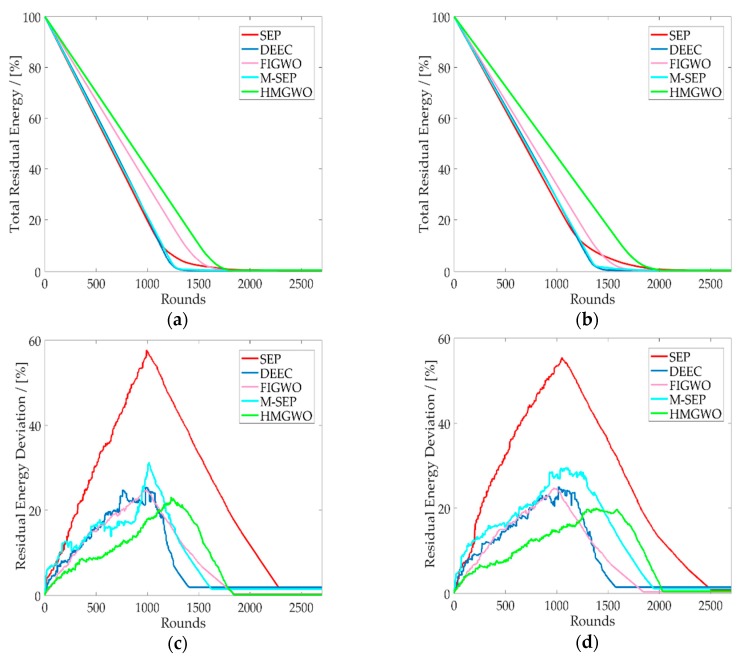
Residual energy relative to the number of rounds. (**a**) Total residual energy of HMGWO against SEP, DEEC, M-SEP, and FIGWO when the ratio of advanced nodes is α=0.1; (**b**) Total residual energy of HMGWO relative to SEP, DEEC, M-SEP, and FIGWO the ratio of advanced nodes is α=0.2; (**c**) The residual energy deviation of HMGWO is compared with SEP, DEEC, M-SEP, and FIGWO when the ratio of advanced node is 0.1; (**d**) The residual energy deviation of HMGWO is compared with SEP, DEEC, M-SEP, and FIGWO when the ratio of advanced node is 0.2.

Figure 5a,b show the total residual energy of the network, from which it is obvious that the HMGWO had a higher total residual energy, which means that the network could survive for a longer period of time. As can be seen in Figure 5c,d, the HMGWO effectively reduced the residual energy deviation compared with other algorithms. In HMGWO, the nodes with more remaining energy were selected as the CH more frequently than the nodes with less remaining energy, so the residual energy deviation was more balanced relative to other protocols. The percentage of the total residual energy and the residual energy difference can be calculated by Equations (19) and (20), respectively.

## 6. Discussion

The HMGWO protocol outperformed the SEP, DEEC, M-SEP, and FIGWO protocols in terms of the network lifecycle, power consumption, and throughput. The reasons are proved and summarized as follows.

The SEP, DEEC, and M-SEP protocols are the routing protocols of HWSNs that do not consider the distance from node to BS when selecting CHs, and they just define the different threshold formulas of CH selection relative to the advanced nodes and normal nodes. In SEP, the selection of CHs in the whole network is inherently random. This makes the distribution of CHs in each round uneven, which will lead to unbalanced energy consumption among nodes and to the premature death of some nodes. Although the high-energy node in SEP has a higher probability of becoming the CH than the ordinary node, due to the random selection of the CHs, the frequency of some high-energy nodes becoming CHs may be small. This results in the slow death rate of the high-energy node, which extends the network instability period and the energy consumption among the nodes is not balanced. When the DEEC protocol selects the CH, the residual energy and the average energy of the network node are taken into account in the weighted election probability. That is to say, when the residual energy of the node is larger than the average energy, the average probability of the node being selected as the CH is higher than that of the node whose residual energy is smaller than the average energy; thus, the energy consumption between advanced nodes and ordinary nodes is reduced, and the life cycle of the network is extended, compared with SEP. In the model of CH selection, compared with DEEC, M-SEP applies the residual energy of nodes and the average energy of network nodes to the threshold formula. However, the weighted election probability does not change. When the residual energy is greater than the average energy, the threshold value of the node is relatively large, but there is still some randomness when the node is selected as the CH. The network lifetime is improved relative to the SEP protocol, but it is not optimized compared to DEEC in terms of residual energy deviation. On the contrary, our algorithm uses the fitness function ($F_{1}$) to select the initial CHs. The fitness function ($F_{1})$ takes into account the residual energy of the node and the distance from the node to the BS, so that the initial CHs are more evenly distributed in the network, and overcomes the randomness of the selected CHs. However, the selection of the initial cluster further fixes and limits the number of CHs, and provides the search range of the MGWO algorithm, thereby reducing the energy consumption in unnecessary clustering, saving network energy consumption, and prolonging the network life cycle.

The GWO is used to select the CHs in FIGWO. When using the GWO algorithm for the selection of CHs, the selection of the final CHs is carried out according to the Euclidean distance (at the end of the iteration, the algorithm can estimate the prey’s position (here, the prey is called a virtual CH), and all nodes will calculate the distance to the virtual CH; the node with the smallest distance will be selected as the CH). In the algorithm proposed in this paper, the final CHs are selected by the fitness function ($F_{2}$), which is designed by the energy of the node and the distance from the node to the virtual CH. The node with high residual energy and nearest distance have a greater probability as the final CH, thereby avoiding the node with low remaining energy to become the final CH, and further reducing the energy consumption deviation of the network. For FIGWO, the GWO is used to select the final CH within each initial cluster, and the selected CH is only optimal in the cluster; however, the set of final CHs may not be optimal throughout the whole network. The FIGWO algorithm only solves the local optimal problem of CHs but does not consider the optimal problem of CHs in the whole network. However, the HMGWO adds a new algorithm to the outer layer of MGWO. The outer layer algorithm selects the bad set of CHs with a certain probability as the initial cluster set of the next iteration, so that the selected CH set is not only the best in the cluster, but also, the final CH set is the best in the whole network. HMGWO makes rational use of the heterogeneity of nodes, solves the randomness of CHs, and considers the global optimality of CHs. Therefore, HMGWO is superior to the SEP, DEEC, M-SEP, and FIGWO protocols in terms of the energy consumption, network life cycle, and throughput.

## 7. Conclusions

In this study, we proposed a routing protocol for HWSNs based on a modified grey wolf optimizer. By defining different fitness functions for both advanced nodes and normal nodes and modifying the GWO, this algorithm can ensure that advanced nodes in the cluster are more likely to be selected as CHs. Consequently, the burden of low-energy nodes being selected as CHs can be reduced, and the network lifetime can be extended. The simulation results show that, compared with traditional SEP, DEEC, M-SEP, and FIGWO protocols, the energy consumption, lifetime, and throughput of the network are significantly improved. In the future, the proposed HMGWO will be extended to larger sensor networks to consider multi-hop communication between CHs, thus reducing energy consumption between a remote BS and CHs. However, when the CHs are selected, how to reasonably reduce the extra energy consumed by all sensor nodes to transmit their positions and energy to the base station, thus reducing the unnecessary energy consumption, will also be a focus of further study.

## Figures and Tables

**Table 1:** Abbreviations used in this paper.

Abbreviations	Explanation
WSN	Wireless Sensor Network
HWSN	Heterogeneous Wireless Sensor Network
CH	Cluster Head
BS	Base Station
GWO	Grey Wolf Optimizer
MGWO	Modified Grey Wolf Optimizer
LEACH	Low Energy Adaptive Clustering Hierarchy Protocol
SEP	Stable Election Protocol
DEEC	Distributed Energy-Efficient Clustering Algorithm
M-SEP	Modified Stable Election Protocol
FIGWO	Fitness-Value-Based Improved Grey Wolf Optimizer
HMGWO	A Routing Protocol for Heterogeneous Wireless Sensor Network Based on the Modified Grey Wolf Optimizer

**Table 2:** Simulation parameters.

Parameter	Value
The area of the sensing region, Number of Sensor Nodes	$100^{2}\left(m^{2}\right),\;N\;=\;100$
Portion of cluster heads	p=0.1
Packet Size	l=4000 bits
Data Aggregation Energy Cost	$E_{DA}=5\;\mathrm{nJ}/\mathrm{bit}$
Energy Cost of Transmitter/Receiver	$E_{elec}=50\;\mathrm{nJ}/\mathrm{bit}$
Transmission Coefficient of Amplifier (free space)	$\varepsilon_{fs}=10\;\mathrm{pJ}/\mathrm{bit}/m^{2}$
Transmission Coefficient of Amplifier (multi-path space)	$\varepsilon_{mp}=0.0013\;\mathrm{pJ}/\mathrm{bit}/m^{4}$
Initial energy of normal node	0.5 J
Ratio of Advanced Node	α=0.1, α=0.2
Weight of Fitness Function	$a_{1}=a_{2}=a_{3}=0.2$
Ratio of Initial Energy of the Advanced Node to that of the Normal Node	β=2
